# The Mechanisms of Soil Conditioner and Switchgrass in Improving Saline–Alkali Soil: A Field Study in a Semi-Arid Area

**DOI:** 10.3390/biology14121788

**Published:** 2025-12-15

**Authors:** Yixuan Li, Qing Liu, Longfei Kang, Kaiyu Zhang, Qiang Li, Feng Ai

**Affiliations:** 1 Shaanxi Key Laboratory of Ecological Restoration in Northern Shaanxi Mining Area, Yulin University, Yulin 719000, China; 2 State Key Laboratory of Soil Erosion and Dryland Farming on the Loess Plateau, Institute of Soil and Water Conservation, Ministry of Water Resources and Chinese Academy of Sciences, Yangling 712100, China

**Keywords:** saline–alkali soil, soil amendment, switchgrass, ecosystem multifunctionality, soil microorganisms

## Abstract

Chemical and plant-based strategies have become increasingly critical for the remediation of saline–alkali soils. However, the underlying mechanisms driving improvements in soil quality and ecological functionality remain insufficiently understood. In this study, we adopted a synergistic remediation approach that integrated multiple switchgrass (*Panicum virgatum* L.) cultivars with a coal-based soil amendment to enhance saline–alkali land. A field experiment was conducted using five switchgrass varieties (YM-1, YM-2, YM-3, YM-4, and YM-5), each receiving a uniform application of the coal-based soil conditioner at 10 t ha^−1^. A traditional control group was not included in this study, as the experimental design focused on direct comparisons between varieties. Our results showed that soil ionic composition played a significant role in shaping microbial activity. Notably, we found that YM-5 treatment exhibited the highest relative soil microbial abundance (22.1%) under the condition of soil amendments. Furthermore, the YM-5 treatment significantly reduced soil Na+ content and exchangeable sodium percentage (ESP) (*p* < 0.05), outperforming other treatments. Compared to YM-2, the YM-5 treatment also resulted in substantial increases in soil organic carbon (SOC) and available potassium (AK), increases of 78.28% and 54.3%, respectively. In addition to enhancing physicochemical parameters, the integration of switchgrass and amendment promoted soil biological vitality. For example, the YM-2 treatment achieved a 7.4% increase in catalase (CAT) activity and a 6.3% reduction in soil pH compared to YM-3, indicating improved redox balance and acid–base regulation. Collectively, these findings provide direct empirical evidence supporting the effectiveness of switchgrass–amendment combinations in saline–alkali soil restoration. Among the tested cultivars, YM-5 demonstrated superior ecological performance and is recommended as the most suitable genotype for saline–alkali soil amelioration when used in conjunction with coal-based amendments.

## Simple Summary

This study explored the synergistic remediation of saline–alkali soils in Northwest China by combining multiple switchgrass (*Panicum virgatum* L.) cultivars with a coal-based soil conditioner. We assessed the soil physicochemical properties and microbial abundance to identify the optimal switchgrass variety for enhancing soil multifunctionality and the ecological utilization potential of degraded saline–alkali lands.

## 1. Introduction

Soil salinization is a globally pervasive challenge, with approximately 20% of arable land reported to be affected to varying degrees. This form of land degradation poses serious threats to global food security and the sustainable use of cultivated land [1]. Saline–alkali soils are characterized by poor permeability, nutrient deficiencies, and limited microbial activity, which collectively constrain crop growth by degrading soil chemical and physical properties. Furthermore, these conditions can adversely affect crops by triggering excessive accumulation of reactive oxygen species (ROS) [2]. Improving the quality of saline–alkali soils has therefore become an urgent and critical priority for safeguarding agricultural productivity and ensuring long-term food security.

In China, saline–alkali soils are primarily distributed across the arid inland regions of the Northwest, the middle and upper reaches of the Yellow River, the Songnen Plain in Northeast China, the Huang-Huai-Hai Plain, and coastal areas along the Bohai Sea coast. According to the 2019 national land survey, saline–alkali areas cover approximately 7.67 million hectares in China [3]. Vast tracts of these lands remain unproductive, making the amelioration and comprehensive utilization of saline–alkali soils a critical national priority. Consequently, the selection and breeding of salt-tolerant plant varieties has become an urgent need [4]. Concurrently, coal gangue, a major industrial solid waste derived from coal chemical processing in the “Ji”-shaped bend of the Yellow River, presents significant challenges. Its resource utilization remains a focal point of research for experts both domestically and internationally. The latest study indicates that coal gangue, rich in inorganic components such as Fe, CaO, and MgO, can effectively mitigate soil compaction, desertification, and salinization. It improves soil permeability, enhances water and nutrient retention capacity, and facilitates better micronutrient uptake via plant roots [5]. While the application of coal-based fertilizers and amendments has been shown to influence soil physicochemical properties [6], reshape microbial community structures [6], and promote plant growth [7], the specific mechanisms driving these effects remain inadequately understood and warrant further investigation.

Currently, for saline–alkali soil remediation, approaches include biological, chemical, and physical methods. For instance, Wang et al. [8] demonstrated that cultivating salt-tolerant crops such as chicory (*Cichorium intybus*) in highly saline–alkali soils significantly reduced soil electrical conductivity while enhancing microbial abundance and nutrient availability, thereby improving soil salinity tolerance and sustainability. Chen et al. [9] focused on increasing carbon storage in topsoil layers by preferentially promoting the accumulation of plant-derived carbon. Zhao et al. [10] significantly enhanced cadmium (Cd) removal efficiency in saline–alkali soils (from 35.1% to 95.1% in the rhizosphere) using fungal-derived nitrogen-doped biochar combined with salt-tolerant bacteria. Zhang et al. [11] reported that phosphorus-containing amendments reduced soil pH from 8.54 to 7.03 and increased alkali-hydrolyzable nitrogen, available phosphorus, and available potassium by 81.68%, 60.31%, and 42.03%, respectively. Gao et al. [12] utilized stabilized sludge products to promote soil aggregate formation, loosening pore space and thereby improving salt leaching capacity. Li et al. [13] found that soil amendments altered soil organic matter composition, accelerating the degradation of O-alkyl C while increasing the sequestration of recalcitrant alkyl C and aromatic C. The gypsum effect in stabilizing sodic dispersing soils includes an increase in the soil electrical conductivity levels to prevent dispersion and reduce ESP [14]. Xing et al. [15] highlighted the critical role of gypsum (CaSO_4_) in reducing exchangeable sodium percentage (ESP), where Ca^2+^ ions displace Na^+^ through cation exchange. Additionally, conventional agricultural practices like irrigation, deep plowing, and topsoil replacement can improve saline soils. However, their implementation is often hindered by high water demand, substantial transportation costs, and incomplete remediation efficacy [16]. It is thus necessary to clearly understand the underlying mechanism and direct evidence for improving soil quality using coal-based solid waste.

Switchgrass (*Panicum virgatum* L.), a highly productive and stress-tolerant C_4_ perennial grass, has demonstrated considerable potential for biomass production. In Georgia, USA, lowland cultivars such as *Alamo* and *Kanlow* have achieved dry matter yields of 16.3 and 15.8 t · ha^−1^, respectively [17]. Fike et al. [18] documented yields under varying management: plots receiving 50 kg Nha^−1^ yielded 11.1 t · ha^−1^, while high-input plots (100 kg N · ha^−1^) yielded 14.9 t · ha^−1^. Despite its high yield and stress tolerance, switchgrass has not been widely applied for saline–alkali soil remediation in China, particularly in the Northwest region. Continuous, efficient, and regionally adapted remediation strategies using switchgrass are notably lacking. A remediation strategy leveraging the synergy between high-yielding, stress-tolerant switchgrass cultivars and soil amendments therefore presents a novel approach. Compared to conventional methods—which are often labor-intensive, costly, and environmentally taxing—this integrated strategy offers superior sustainability and ecological resilience.

In this study, saline–alkali soil in Northwest China was used as a test field. Our scientific hypotheses are as follows: (i) The combined use of switchgrass and amendments effectively improves the physicochemical properties of saline–alkali soil; (ii) Switchgrass–amendment treatments influence the structural diversity of soil microbial communities and enhance their correlations with key environmental factors. This study aimed to identify the switchgrass (*Panicum virgatum* L.) cultivars that, when combined with soil conditioners, exhibit the most effective synergistic improvement of saline–alkali soils, thereby providing robust technical support and practical applications for the restoration of degraded saline–alkali land. This study holds significant promise for overcoming the current technical bottlenecks in saline–alkali soil remediation in Northwest China, providing an advanced and innovative pathway for efficient soil restoration.

## 2. Materials and Methods

### 2.1. Study Site Description

**Figure 1:**
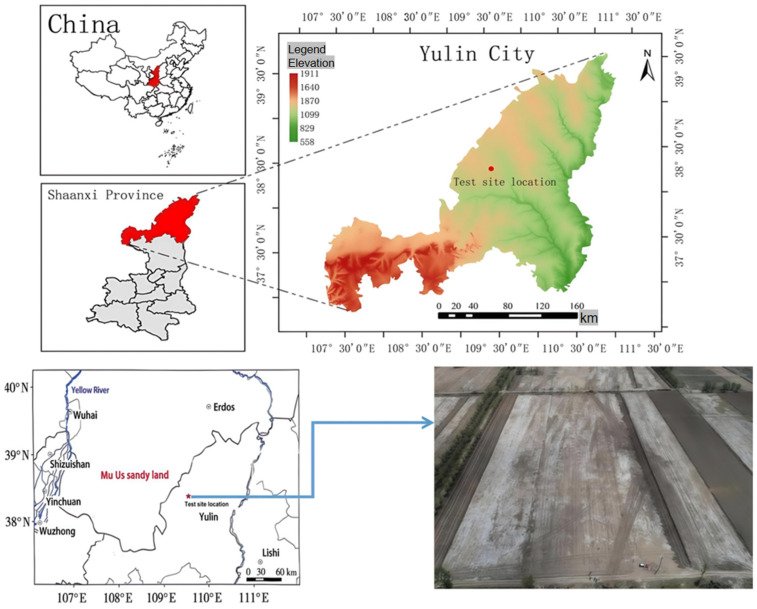
Study area.

The field experiment was conducted at Huangshaqidun Village, Qinhe Town, Yuyang District, Yulin City, Shaanxi Province, China (109°54′84″ E, 38°20′57″ N; elevation 1131 m). The site experiences a semi-arid continental climate characterized by the following: mean annual precipitation, 414 mm (predominantly concentrated from July to October); mean annual temperature, 10.1 °C; mean annual frost-free period, 151 days; mean annual solar radiation, 139.23 kJ · cm^−2^ (equivalent to 1392.3 mJ · m^−2^); And mean annual sunshine duration, 117 days Baseline soil properties (0–20 cm depth) prior to treatment application were as follows: soil organic carbon (SOC), 5.09 g · kg^−1^; alkali-hydrolyzable nitrogen (AN), 18.07 mg · kg^−1^; available phosphorus (AP), 14.46 mg · kg^−1^; available potassium (AK), 67 mg · kg^−1^; total soluble salts (TSSs), 2.31 g · kg^−1^; and soil pH (1:2.5 soil/water), 8.90. These properties classify the soil as slightly saline–alkali [19] (see Table S1 and Figure 1).

### 2.2. Experimental Materials

The field experiment was conducted in August 2024 on saline–alkali soil within the semi-arid climate zone of the Loess Plateau, China. This study employed coal gangue and five switchgrass (*Panicum virgatum* L.) varieties as remediation materials.

### 2.3. Experimental Design

A randomized complete block design was implemented. Treatments comprised five switchgrass cultivars: YM-1, YM-2, YM-3, YM-4, YM-5 (Table S2). A soil conditioner was applied uniformly at 10,000 kg·ha^−1^ (equivalent to 10 t·ha^−1^), and its composition is detailed in Table S3.

### 2.4. Soil Conditioner Composition and Panicum virgatum L. Cultivation

Coal gangue was produced by the Baojucai Coal Gangue and Coal Sludge Comprehensive Utilization Co., Ltd(The company is located in Yulin City, Shaanxi Province, China). An S-type sampling method was used, and the coal gangue comprised 1.1 mg · kg^−1^ of available iron, 1.4 mg · kg^−1^ of available phosphorus, and 16.0 mg · kg^−1^ of available potassium and exhibited a pH value of 9.3. The coal-based soil conditioner was produced using 78.0% of coal gangue, 15.0% manure, and 7.0% of flue gas desulfurization gypsum (FGD gypsum) (Table S3) and was then applied to the soil surface (0–20 cm), and the top 20 cm of soil was thoroughly mixed using a rotary tiller. Each plot size is 10.0 × 1.0 m (10.0 m^2^), and the number of replicates per treatment is three.

The switchgrass was seeded at a rate of 7.0 kg · hm^−2^ according to the plant species property, and the row spacing was 0.3 m. Planting depth was 1–2 cm under the soil surface. Sprinkler irrigation was used throughout the experiment at the rate of once per month.

### 2.5. Sample Collection

Seedlings can be observed 15 days after sowing. Sampling was performed three times (18 July, 18 August, and 18 September in 2024). In this experiment, a rotary tiller was used to mix the soil with gangue soil conditioner, with a tillage depth of 0–10 cm. To ensure the rigor of the experiment, a soil drill (diameter = 1.0 cm, length = 50 cm) was employed to collect surface soil samples (0–20 cm) [20]. A serrated knife was used to remove gravel and plant residues. For each treatment plot, the S five-point method was used for sampling, and after the sampling was completed, the 5 sub-samples were mixed into a single mixed sample to ensure uniformity. Each mixed soil sample was divided into two parts, one part of which was used for soil nutrient determination (i.e., available phosphorus, soil salt solubility, alkali hydrolyzable nitrogen, available phosphorus, and available potassium) and physicochemical property determination through a 0.15 mm screen. The other part of the soil sample was passed through a 2.00 mm sieve and stored at 4.0 °C for soil enzyme activity determination. One part of the plant samples was used to determine the fresh weight, and the other part was dried at 105 °C for 30 min and then at 75 °C to determine the dry weight. Plant samples were ground and sieved (0.5 mm), and other properties were measured.

### 2.6. Analytical Methods

Soil properties were determined as follows: moisture content (MC) via gravimetric oven-drying [21]; alkali-hydrolyzable nitrogen (AN) via alkaline diffusion [22]; available potassium (AK) extracted with ammonium acetate (NH_4_OAc) and determined using a flame photometer [23]; soil organic carbon (SOC) using potassium dichromate oxidation (external heating [24]); available phosphorus (AP) via the Olsen bicarbonate method; and pH potentiometrically in 1:2.5 soil/water suspension (PHSJ-4F pH meter, Leici, Shanghai City, China). Microbial communities were characterized via Illumina-based 16S rDNA amplicon sequencing [25] of composite samples from paired column locations per plot. Exchangeable Na^+^ was quantified via NH_4_OAc-NH_4_OH flame photometry, while Ca^2+^ and Mg^2+^ were analyzed via EDTA titration [26]. Cation exchange capacity (CEC) was measured via sodium acetate saturation-flame photometry, and exchangeable sodium percentage (ESP) derived from Na^+^/CEC ratios. Soil enzyme activities included sucrase (SUC) with 3,5-dinitrosalicylic acid colorimetry; catalase (CAT) via KMnO_4_ titration; alkaline phosphatase (ALP) using disodium phenyl phosphate-4-aminoantipyrine; and polyphenol oxidase (PPO) via standard colorimetry.

Total alkalinity, sodium adsorption ratio (*SAR*), and exchangeable sodium percentage (*ESP*) were calculated according to established methodologies by Gao et al. [27] and Yu et al. [15] as follows (Equations (1)–(3)):

(1) $$Total\;Alkalinity=[{CO}_{3}^{2-}]+[{HCO}_{3}^{-}]$$

(2) $$SAR=\frac{Na^{+}}{\sqrt{(Ca^{2+}+Mg^{2+})/2}}$$

(3) $$ESP=(Exchangeable\;{Na}^{+}/CEC)\times 100$$

### 2.7. Data Processing

Data were analyzed using one-way ANOVA followed by post hoc testing with Tukey’s HSD in SPSS 24.0 (IBM, Armonk, NY, USA) to determine significant differences (*p* < 0.05) among switchgrass varieties. The relationships between various properties were assessed through Pearson correlation analysis. Quantitative results are expressed as mean ± standard deviation (SD). Redundancy analysis (RDA) was carried out to explore the effects of switchgrass cultivation on soil properties and soil bacterial communities using Canoco 4.5 software (Microcomputer Power). Comprehensive evaluation of saline–alkali soil remediation efficacy was performed using weighted membership function values [28] with all figures generated in Origin 2023 (v10.0.1.178, OriginLab Corporation, Northampton, MA, USA). The membership function equation is as follows:

(4) R(xi)=(Xi−Xmin)/(Xmax−Xmin) 

The inverse membership function analysis equation is as follows:

(5) R(xi)=1−(Xi−Xmin)/(Xmax−Xmin)

where *R*(*xi*) is the measured value of the index, and *Xmax* and *Xmin* are the maximum and minimum values of a certain index for all tested materials, respectively.

Moreover, in order to further comprehensively evaluate the improvement effect of the amendment on the soil, soil quality index was calculated according to the following method: correlation-based weights (*Wci*): In this approach, indicators are weighted according to their interrelationships (Table S3) [29]. Firstly, the correlation coefficient matrix table of all soil indexes was calculated. The correlation coefficient (*Si*) of a single indicator with all other indicators is then summarized (Equation (6)). Then dividing the sum by the maximum value (*Wi*). The weight calculation expression is as follows (Equation (7)). The soil quality index (*SQI*) is calculated as follows (Equation (8)). The value *n* is the number of indicators.

(6) $$Si={\sum \;}_{i=1}^{n}.Ci$$

(7) Wci=Si/Smax

(8) SQI=∑(Wi×Si)

## 3. Results and Analysis

### 3.1. Soil Physicochemical Properties

Significant varietal effects of switchgrass on soil properties were observed under coal gangue-based amendments (Table 1). Specifically, cultivars YM-1 and YM-2 exhibited significantly lower soil *Na^+^* content compared to other varieties; for example, the content of *Na^+^* in YM-1 treatment was 82.31% lower than in the YM-4 treatment. YM-2 exhibited a markedly elevated soil moisture content (MC), 53.4% higher than YM-1 (*p* < 0.05), and concurrently reduced soil pH by 8.8% relative to YM-1. Cultivar YM-5 yielded significantly higher soil ammonium nitrogen (*AN*) and available potassium (*AK*) values than the other varieties, registering increases of 46.52% and 63.49%, respectively, compared to the lowest values observed in YM-1. The soil organic carbon (SOC) content in YM-5 treatment was significantly higher in than other treatments; for instance, it increased by 78.28% relative to the YM-2 treatment. In contrast, soil available phosphorus (AP) was significantly elevated in YM-3 compared to other cultivars, with a 67.58% increase relative to YM-1.

**Figure 2:**
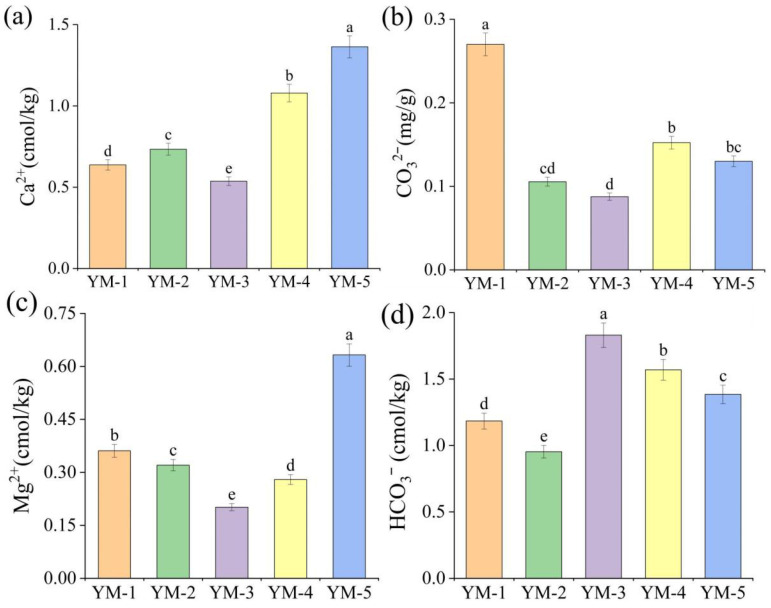
Effects of different treatments on the concentrations of Ca^2+^ (**a**), CO_3_^2−^ (**b**), Mg^2+^ (**c**), and HCO_3_^−^ (**d**). Lowercase letters indicate whether there are significant differences between samples of the same indicator (The a b c d e is difference was significant. *p* < 0.05).

Soil cation concentrations varied significantly among switchgrass cultivars (Figure 2). While Ca^2+^ and Mg^2+^ averaged 174.39 mg/kg and 43.49 mg/kg, respectively, mean HCO_3_^−^ and CO_3_^2−^ concentrations were 0.99 mg/kg and 0.05 mg/kg. Cultivars differentially influenced Na^+^ in saline–alkali soil: YM-2 consistently exhibited significantly lower Na^+^ and significantly higher soil moisture content (*MC*) than other cultivars, indicating superior water retention capacity. In contrast, YM-3, YM-4, and YM-5 had significantly higher *Na^+^* than the overall mean. Soil Ca^2+^ ranged from 106.51 to 274.05 mg/kg; YM-3 contained significantly lower Ca^2+^ (107.63 mg/kg), while YM-5 contained significantly higher Ca^2+^ (273.32 mg·kg^−1^) than other cultivars. For anions, HCO_3_^−^ in YM-2 (0.56 mg/kg) was the lowest than other treatments. Moreover, the YM-3 treatment obtained the highest HCO_3_^−^ content among various treatments, which showed a 91.98% increase relative to the YM-2 treatment. CO_3_^2−^ concentrations in YM-2, YM-3, and YM-5 were lower than that in the YM-3 treatment (0.03 mg/kg).

### 3.2. Soil Enzyme Activities

**Figure 3:**
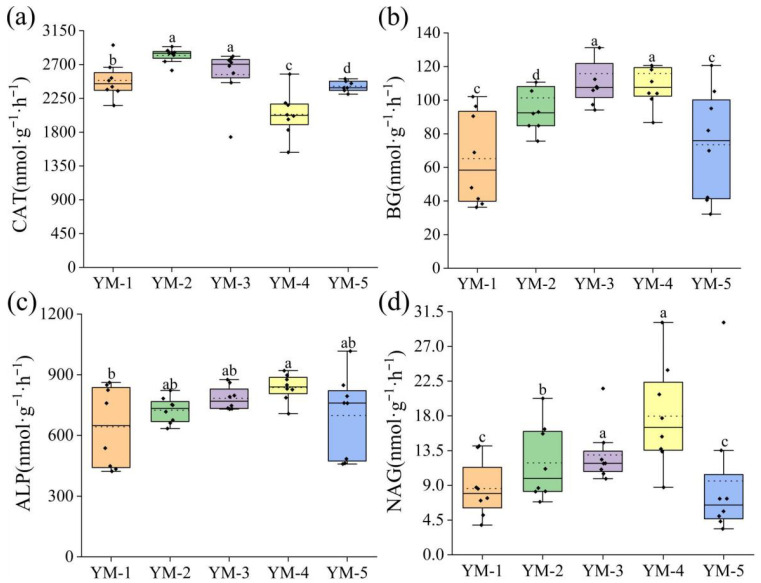
Changes in soil enzyme activity under different varieties. Changes in soil catalase (CAT) (**a**), β-glucosidase (BG) (**b**), alkaline phosphatase (ALP) (**c**), and β-1.4N-acetylglucosidase (NAG) (**d**) under different varieties (The a b c d is difference was significant. *p* < 0.05).

Soil enzyme activity serves as a key indicator of saline–alkali land improvement efficacy. Among the five switchgrass cultivars (Figure 3), YM-4 treatment exhibited higher activity levels for β-glucosidase (BG), alkaline phosphatase (ALP), and β-1,4-N-acetylglucosaminidase (NAG) (Figure 3). Catalase (CAT) activity was significantly higher in YM-2 and YM-3 than the other treatments. In addition, YM-2 treatment showed the highest CAT activity. The activities of BG, ALP, and NAG obtained similar trends, consistently demonstrating higher values in YM-4. The difference in β-glucosidase (BG) activity between YM-3 and YM-4 was minimal (1.33%). YM-2 and YM-3 exhibited significantly higher catalase (CAT) activity compared to other treatments. In addition, YM-2 treatment showed the highest CAT activity (*p* < 0.05). Across the enzymatic profile of β-glucosidase (BG), alkaline phosphatase (ALP), and N-acetyl-β-glucosaminidase (NAG), YM-5 registered the lowest NAG activity yet outperformed YM-1 in both BG and ALP. Concurrently, YM-4 achieved the highest activities for all three enzymes, significantly exceeding those of every other treatment (*p* < 0.05).

### 3.3. Comprehensive Evaluation of Soil Properties

**Figure 4:**
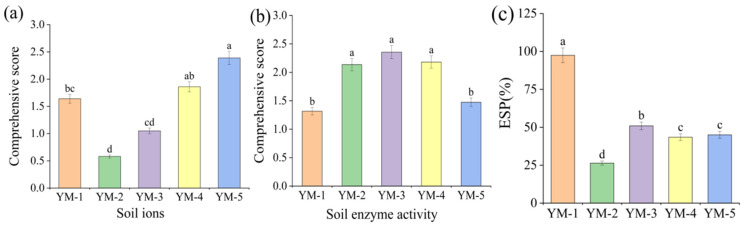
Different comprehensive evaluations of soil ion (**a**), soil enzyme activity (**b**), and soil ESP (**c**) content of the treatment, with lowercase indicating whether there were significant differences between samples with the same index (The a b c d is difference was significant. *p* < 0.05).

Soil exchangeable sodium percentage (*ESP*) measured at harvest varied significantly among treatments (Figure 4). The highest *ESP* (97.45%) occurred in the YM-1 control group. Cultivated treatments exhibited lower *ESP* values ranging from 26.40% to 50.94%, with YM-2 showing the lowest value (26.40%). Across cultivars, *ESP* followed the descending order: YM-1, YM-3, YM-5, YM-4, YM-2.

A comprehensive evaluation of soil salinity control efficacy was conducted using the inverse membership function for key ions *Na^+^*, *Ca*^2+^, *HCO*_3_^−^, *CO*_3_^2−^, and *Mg*^2+^ (Figure 4). Lower composite scores indicate superior salinization mitigation. Analysis revealed YM-5 yielded the least favorable score (2.39), signifying the weakest performance in soil salinity control among cultivars.

Composite scores for soil enzyme activity catalase, β-glucosidase, alkaline phosphatase, β-1,4-N-acetylglucosaminidase were calculated using membership function analysis (Figure 4), where higher values indicate superior soil amelioration efficacy. The comprehensive evaluation identified YM-3 as the top-performing cultivar with a score of 2.36.

### 3.4. Soil Microbial Communities

**Figure 5:**
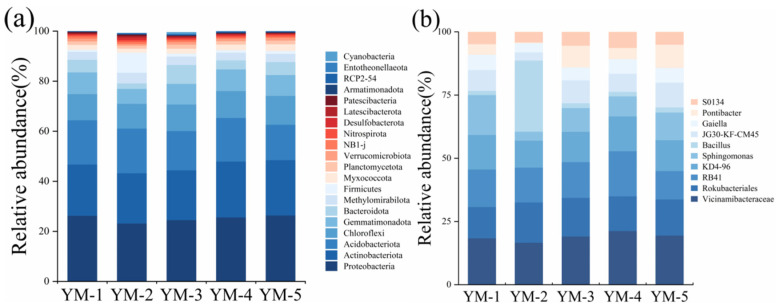
Statistics of soil microorganisms at phylum (**a**) and genus levels (**b**).

Bacterial community composition differed significantly among switchgrass cultivars in saline–alkali soil (Figure 5a). *Proteobacteria* dominated across all treatments (23.05–26.89%), with the highest proportion observed in YM-5 (26.37%). YM-5 treatment exhibited the highest relative abundance (26.37%), there was a 14.06% increase compared to YM-2 treatment. Additionally, the abundance of *Proteobacteria* in YM-5 was similar to that in YM-1 treatment, which exhibited only a 0.61% difference. Subdominant phyla included *Actinobacteriota*, *Acidobacteria*, *Chloroflexi*, *Gemmatimonadetes*, *Bacteroidetes*, *Methylomirabilota*, and *Firmicutes*. Notably, YM-5 showed the highest *Actinobacteriota* abundance (22.11%), suggesting synergistic effects with soil conditioners in shaping dominant microbial communities. YM-4 exhibited elevated abundance in both *Acidobacteria* and *Chloroflexi* relative to other cultivars.

At the genus level (Figure 5b), YM-2 exhibited significantly higher relative abundance of *Bacillus* than other cultivars. *Vicinamibacteracea e* emerged as the dominant bacterial group across all treatments (16.56–21.21%), peaking in YM-2 (21.21%). Subdominant genera included *RB41*, *Rokubacteriales*, *KD4-96*, and *Sphingomonas*, collectively forming the core microbiome across the five cultivars.

### 3.5. Biological and Non-Biological Factors Contributing to Changes in Soil Quality

**Figure 6:**
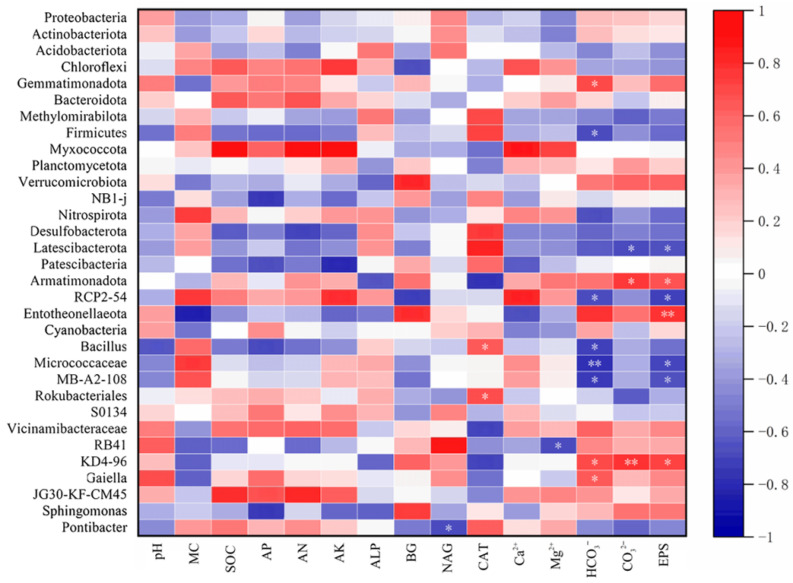
Correlation analysis between microbial phylum and genus species abundance with soil physicochemical properties and plant physiological indexes, with * and ** at the significant levels of *p* < 0.05 and *p* < 0.01, respectively.

Analysis of correlations between microbial communities and 15 environmental factors (Figure 6) revealed significant relationships within the soil system. The dominant soil microflora exhibited positive correlations with soil moisture content and pH. At the phylum level, *Myxomycota* showed significant positive correlations with soil Ca^2+^, Mg^2+^, alkali hydrolyzable nitrogen (*AN*), organic matter (*OM*), and available potassium (*AK*). *Verrucomicrobiota* was positively correlated with soil β-glucosidase (*BG*) activity. Conversely, *Desulfobacterota* displayed significant negative correlations (*p* < 0.01) with soil *OM*, available phosphorus (*AP*), *AN*, and *AK*. Examining dominant microflora correlations at the community level confirmed significant positive associations with soil pH, *OM*, *AP*, *AN*, and *AK*, alongside a negative correlation with catalase (*CAT*) activity. At the genus level, *Bacillus* correlated positively with soil moisture and *CAT*, but negatively with *AP*, *AN*, and HCO_3_^−^. Finally, RB41 demonstrated significant negative correlations (*p* < 0.01) with soil moisture, *OM*, and *AN*, while correlating positively with β-1, 4-N-acetylglucosaminidase (*NAG*) activity.

**Figure 7:**
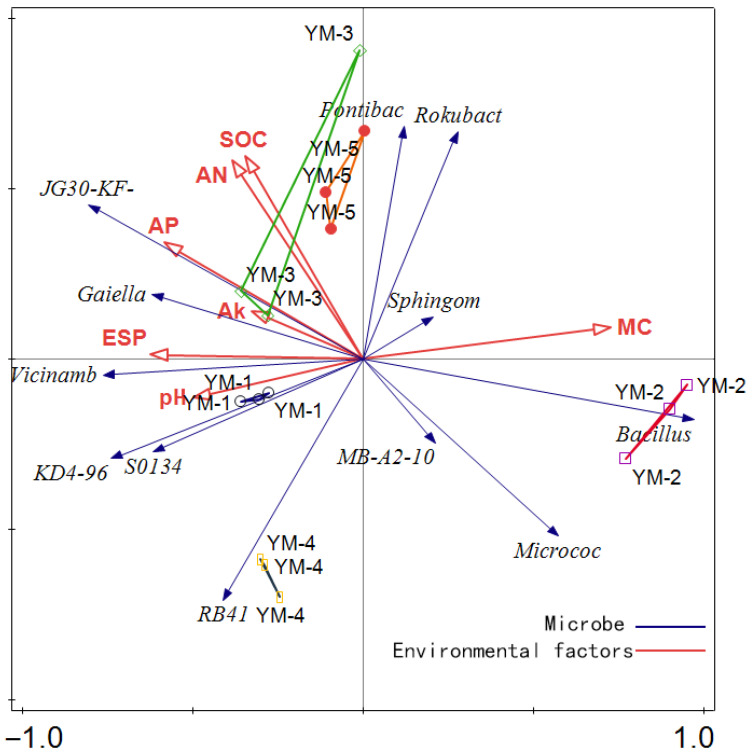
Redundancy analysis (RDA) of soil physicochemical properties and biological factors under soil amendment with soil conditioner and switchgrass. The red and blue arrows in the figure represent soil physicochemical indicators and soil biological factors, respectively.

Redundancy analysis (RDA) elucidated relationships between soil microbial communities and environmental factors (Figure 7). Microbial communities and environmental factors were distributed primarily across the first, second, and fourth quadrants, indicating limited overall association among these indicators. The first two RDA axes (Figure 7) explained 82.56% (Axis 1: 82.56%) and 6.98% (Axis 2: 6.98%) of the environmental factor variance. Separately, RDA of microbial communities (Figure 7) showed the first two principal axes cumulatively explained 53.03% of community variation (Axis 1: 34.72%, Axis 2: 18.31%). The YM-2 switchgrass treatment exhibited distinct clustering from other treatments and demonstrated a strong association with soil moisture content (MC) and *Bacillus* spp. abundance, suggesting this treatment favored *Bacillus* proliferation and moisture retention. Conversely, treatments YM-1, YM-3, and YM-5 showed strong correlations with key soil factors: soil organic carbon (*SOC*), alkali hydrolyzable nitrogen (*AN*), available phosphorus (*AP*), exchangeable sodium percentage (*ESP*), and pH. Furthermore, YM-3 and YM-5 were positively associated with genera such as *Fontibacter* and *Rokubacteria*. Combined with the specific association of YM-2 with *Bacillus*, these results indicate that different switchgrass varieties selectively enriched distinct microbial taxa and influenced specific soil properties.

A composite soil quality index (SQI), integrating key physicochemical properties and enzyme activities, was used for comprehensive soil evaluation. As presented in Table 2, treatment YM-5 achieved the highest SQI score (5.477), followed in descending order by YM-4 (4.214), YM-3 (4.018), YM-1 (2.702), and YM-2 (2.245). This ranking demonstrates that the YM-5 switchgrass variety combined with soil conditioner provided the most effective amelioration of saline–alkali soil conditions.

**Figure 8:**
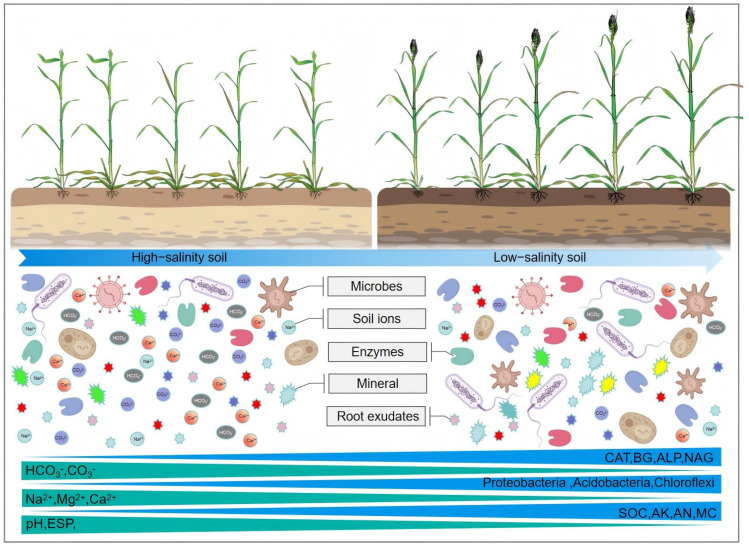
Mechanism of improving saline–alkali soil through the coupling of switchgrass and soil conditioner. Colored circles in figure represent different ions, while other unlabelled shapes represent different types of root exudates.

The combined use of switchgrass varieties and soil conditioners enhanced saline–alkali soils by boosting microbial abundance and facilitating ion transport via root exudates [3,30]. This enhancement improved the soil’s physicochemical properties, which in turn led to successful land rehabilitation (Figure 8).

## 4. Discussion

### 4.1. Effect of Switchgrass Combined with Coal-Based Conditioner on Soil Properties

Soil conditioners are a critical strategy for saline–alkali soil remediation globally. This study demonstrates that combining switchgrass (*Panicum virgatum* L.) varieties with amendments significantly enhances soil quality. Specifically, the YM-5 variety exhibited the highest soil enzyme activity score, attributable to root exudates stimulating microbial activity [31]. Amendments significantly improved soil physicochemical properties [32], facilitating the conversion of organic phosphorus to available phosphorus (AP). This enhanced AP bioavailability promotes crop growth and yield [33,34]. Organic amendments similarly reduce soil pH, electrical conductivity, and salt ions while increasing cation exchange capacity [35]. The composite soil quality index (SQI), integrating physicochemical and enzymatic indicators, confirmed YM-5′s superiority with a score of 5.477. Furthermore, soil microbiota key quality indicators respond to amendments through enhanced enzyme activity linked to yield increases [36]. Interactions between soil properties and nutrients create dynamic environments that shape microbial communities and suppress plant pathogens [37]. Consequently, our amendment application elevated saline soil enzyme activity and microbial abundance. Notably, Cai et al. [38] observed that organic amendment ratios stimulate seedling growth and rhizosphere microbiomes in saline soils, though groundwater depth modulates nutrient availability and plant uptake rates.

The integrated remediation of saline–alkali soils using *Panicum virgatum* varieties and soil conditioners significantly altered microbial community diversity and environmental factor relationships. Dominant phyla were *Proteobacteria* (24.39% relative abundance), *Actinobacteria*, and *Acidobacteria* (16.51–24.39%), consistent with established agricultural soil profiles [39,40]. While switchgrass variety had minimal influence on overall phylum-level dominance-suggesting inherent community stability-significant taxon-specific shifts occurred. Notably, YM-5 and YM-4 preferentially enriched *Acidobacteria* and *Chloroflexi* versus other varieties. This phylum-specific selection demonstrates that plant–amendment combinations shape distinct microbial consortia in saline systems, potentially through root exudate-mediated rhizosphere pH modulation [41]. Such restructuring enhances plant–microbe interactions that improve stress resilience and nutrient cycling efficiency [42], while amendments further enhanced soil microbial coverage [32]. Critically, plant variety significantly affected soil moisture content (SMC; * *p* * < 0.05), with YM-2 exhibiting 114.59% higher SMC than YM-1, confirming switchgrass’s role in soil stabilization and hydrological regulation [43].

### 4.2. Response of Switchgrass Synergistic Conditioner to Soil Microbial Communities

Under saline–alkali stress, Na^+^ enters plant cells via voltage-independent channels (VICs), sodium-permeable K^+^ inward-rectifying channels (KIRCs), and high-affinity K^+^ transporters (HKTs), thereby disrupting K^+^ homeostasis through reduced cytosolic accumulation and enhanced efflux [44]. Concurrent Na^+^/K^+^ competition due to shared physicochemical properties elevates cytoplasmic Na^+^/K^+^ ratios, impairing metabolic processes [45]. These physiological adaptations enable plants to directly modify soil physicochemical properties while indirectly enhancing microbial abundance via improved soil water retention. Consequently, switchgrass salt-tolerance mechanisms demonstrate significant phylum-level correlations with soil microbiota composition, consistent with Yang et al. [46].

Soil pH is widely recognized as a primary driver of bacterial community composition and diversity, simultaneously regulating microbe-mediated organic matter decomposition processes. Alterations in soil pH directly influence microbial energy metabolism, thereby altering the dynamics of nutrient release into the soil by microbial communities. For instance, bacterial growth rates declined fivefold following a pH decrease from 8.3 to 4.5 [47]. The optimal pH range for microbial activity and diversity spans 6 to 8 [48], while most enzymes achieve peak activity between pH 4.5 and 7. Consequently, the most pronounced short-term changes in soil organic matter decomposition driven by inputs of fresh organic matter or sustained nutrients are anticipated at approximately pH 6 [49].

At a soil pH of 7.75, the YM-5 switchgrass variety exhibited enhanced performance, characterized by significantly increased plant height, elevated CO_2_ concentration, and a higher net photosynthetic rate than other varieties. This correlated with markedly raised levels of Mg^2+^ and Ca^2+^ ions. This study postulates that the increase in Mg^2+^ ions mitigates nickel toxicity, a mechanism that ultimately results in enhanced dry matter accumulation [50]. According to Zhang et al. [51] biochar mitigates soil sodicity in saline–alkali soils by providing sufficient Ca^2+^ to promote sodium leaching, which effectively reduces soil sodium content.

*Bacillus* relative abundance was significantly higher in the YM-2 switchgrass variety compared to others, in which it was non-dominant. This disparity is likely attributable to stimulation of soil *Bacillus* populations by YM-2 root exudates, amplified by organic matter from the soil conditioner, resulting in significantly enhanced abundance [52]. *Vicinamibacteraceae* was consistently dominant across all five treatments. Variations in the relative abundances of other bacterial groups were more pronounced among less abundant taxa. Our analysis revealed significant positive correlations (*p* < 0.05) between *Vicinamibacteraceae* relative abundance and key soil properties: organic matter content, pH, available *p*, available K, alkali-hydrolyzable N, and *HCO*_3_^−^ concentrations. A significant negative correlation was detected with soil catalase activity. Collectively, these findings indicate that shifts in soil physicochemical properties, acting synergistically with switchgrass presence and varietal differences in ecological adaptation to environmental stress, shape symbiotic relationships among soil microorganisms, an interpretation supported by Wu et al. [53].

Soil Na^+^ concentration in YM-2 was significantly lower than in other switchgrass varieties across all treatments, while simultaneously exhibiting the highest soil moisture content (MC). This indicates that the YM-2 variety provides the most pronounced improvement in water retention within saline–sodic soils among the tested varieties. Consequently, soluble salt ions in YM-2 soil varied with soil moisture, and CO_3_^2−^ levels remained consistently lower compared to other varieties. These observations demonstrate that CO_3_^2−^, Na^+^, and HCO_3_^−^ ions are strongly influenced by soil water content and exhibit high mobility, consistent with findings on organic amendment remediation of saline–sodic soils by Cui et al. [54]. Liang, F. et al. [55] demonstrated that tomato roots absorbed 43.8% of HCO_3_^−^ from the soil for plant metabolism, thereby sequestering HCO_3_^−^ in plant tissues after soil amendment using organic matter fertilizer. Meanwhile, the enhanced HCO_3_^−^ alleviated the limitation of carbon supply for nitrogen assimilation in the root system, thereby improving the impact of salinity on hydroponic tomato plants [56]. Moreover, about 44.4% of the HCO_3_^−^ synthesized by root microbiota was re-secreted into the soil, contributing to soil organic carbon formation, while 5.5% remained in the soil as insoluble compounds, indirectly enhancing soil carbon sequestration capacity.

Na^+^ is the most abundant soluble cation in plants. It enters plant roots via ion channels and transporter proteins during water and nutrient uptake. Transpiration drives water loss but concentrates Na^+^ and other ions within plant tissues [57]. Saline–sodic soils are typically characterized by low organic matter content, exchange sites dominated by Na^+^, limited retention capacity for other cations, and consequently low cation exchange capacity (CEC) [58].

Soil salinity and exchangeable sodium percentage (ESP) exhibited inverse trends in YM-4 and YM-5. This discrepancy may arise because elevated salt concentrations in the soil solution can suppress Na^+^ exchange reactions [59]. Soluble Ca^2+^ plays a critical role by facilitating Na^+^ displacement and salt leaching, thereby reducing soil ESP [60]. As Abbas et al. [61] demonstrated, Ca^2+^ and Mg^2+^ displace non-plant-available Na^+^ from soil colloids through cation exchange. Subsequent leaching via groundwater movement, rainfall, or irrigation then reduces Na^+^ adsorption rates. The study revealed that in the YM-1 variety, a soil exchangeable sodium percentage (ESP) range of 96.50–98.11 resulted in the lowest observed activities of the enzymes β-glucosidase (BG), β-1,4-N-acetylglucosaminidase (NAG), and alkaline phosphatase (ALP). Concurrently, levels of soil organic carbon (SOC), available phosphorus (AP), alkali-hydrolyzable nitrogen (AN), and available potassium (AK) also reached their minima. This co-occurrence of biological and nutritional decline, coupled with an ESP exceeding 15% (in contrast to the more tolerant YM-5 variety), likely contributed to the degradation of soil structure and hydraulic properties [62]. The composition of fungal community and bacterial diversity contributed the most to SQI, while the increase in soil nutrient level or the indirect change in microbial community affected SQI [63].

## 5. Limitations and Implications

This study demonstrates that the combination of coal gangue soil amendments and different switchgrass cultivars synergistically improves saline–alkaline soil by enhancing soil quality and altering bacterial community diversity, thereby increasing soil nutrient availability. However, several limitations should be noted: (i) When evaluating the effects of coal gangue amendments on soil quality and plant productivity, future studies should consider complete soil profiles including the spatial scale of the plow layer (>20 cm). (ii) Control treatment (CK) (such as soil amendments alone or switchgrass alone) should be included to quantify their individual contributions. (iii) Dynamic data capturing the interannual (long-term) effects of switchgrass on soil should be incorporated to better understand temporal variations. (iv) The composition of coal gangue amendments (mineral composition, toxic substances) may vary significantly due to differences in raw materials and production processes, which could negatively affect soil–plant systems and even cause environmental pollution. Real-time monitoring is essential to assess the stability and environmental risks of these amendments in soil quality improvement.

## 6. Conclusions

This study implemented an integrated phyto-microbial and chemical amendment strategy, leveraging switchgrass’s (*Panicum virgatum* L.) high biomass yield and saline–sodic tolerance alongside soil conditioners that enhance microbial communities, improve soil physicochemical properties, and reduce pH in degraded saline–sodic soils. Our results revealed a significant positive correlation between varietal selection under soil conditioner application and elevated soil enzyme activity. Critically, rigorous assessment via the soil quality index (SQI) and membership function analysis (MFA) identified YM-5 co-applied with soil conditioner as the optimal treatment, demonstrating superior efficacy for saline–sodic soil reclamation. This synergistic approach achieves simultaneous soil amelioration and ecosystem restoration, establishing a scientifically grounded pathway for high-value utilization of salt-affected lands.

## Figures and Tables

**Table 1:** Basic properties of switchgrass varieties (a,b,c,d,e The difference was significant. The * and ** at the significant levels of *p* < 0.05 and *p* < 0.01, respectively).

Soil Indicators	YM-1	YM-2	YM-3	YM-4	YM-5	*p*-Value
pH	8.13 ± 0.05 a	7.62 ± 0.11 b	8.10 ± 0.13 a	8.11 ± 0.12 a	7.75 ± 0.09 b	0.047 *
AN (mg/kg)	38.3 ± 0.37 c	44.5 ± 0.34 d	42.1 ± 0.34 b	39.6 ± 0.34 c	59.2 ± 0.72 a	0.015 *
AK (mg/kg)	44.4 ± 0.31 d	65.5 ± 0.28 d	50.5 ± 2.58 c	63.1 ± 0.11 b	72.5 ± 0.86 a	0.023 *
AP (mg/kg)	17.1 ± 0.13 c	24.1 ± 0.32 d	28.7 ± 0.68 a	23.2 ± 0.28 b	23.5 ± 0.32 b	0.026 *
MC (%)	4.25 ± 0.19 e	9.12 ± 0.08 a	5.41 ± 0.18 d	6.72 ± 0.13 c	8.58 ± 0.23 b	0.039 *
SOC (g/kg)	7.63 ± 0.25 d	6.40 ± 0.25 e	10.7 ± 0.17 b	9.67 ± 0.06 c	11.4 ± 0.09 a	0.008 **
Na^+^ (cmol/kg)	0.287 ± 0.66 c	0.254 ± 0.27 c	1.01 ± 0.46 b	1.44 ± 85.9 a	1.14 ± 1.57 b	0.042 *

**Table 2:** Standard deviation, weight, and comprehensive scores for soil physical and chemical indexes under different switchgrass cultivars.

Dispose	pH	MC	SOM	AP	AN	Ak	ALP	BG	NAG	CAT	Ca^2+^	Mg	HCO^3−^	CO_3_^2−^	Soil Quality
YM-1	0.922	0.000	0.112	0.125	0.787	0.000	0.000	1.000	0.376	0.401	0.121	0.370	1.000	1.000	2.702
YM-2	0.084	1.000	0.000	0.000	0.000	0.031	0.745	0.089	0.512	1.000	0.239	0.276	0.000	0.111	2.245
YM-3	1.000	0.237	0.387	1.000	1.000	0.21	0.931	0.059	0.560	0.933	0.000	0.000	0.632	0.000	4.018
YM-4	0.922	0.506	0.297	0.778	0.857	0.666	1.00	0.142	1.000	0.000	0.656	0.182	0.448	0.370	4.214
YM-5	0.000	0.887	1.000	0.787	3.107	1.000	0.293	0.000	0.000	0.481	1.000	1.000	0.322	0.259	5.477
Weight	0.854	1.000	0.904	0.531	0.611	0.576	0.601	0.535	0.320	0.407	0.236	0.065	0.261	0.137	/

## Data Availability

Dataset available on request from the authors.

## Supplementary Materials

The following supporting information can be downloaded at: https://www.mdpi.com/article/10.3390/biology14121788/s1, Table S1: Classification standards of saline–alkali soil; Table S2: Basic properties of soil conditioners; Table S3: Soil physicochemical indexes of different switchgrass varieties (*p* < 0.05).
