# Supplementary material for: The Mechanisms of Soil Conditioner and Switchgrass in Improving Saline–Alkali Soil: A Field Study in a Semi-Arid Area
Source: Biology (Basel). 2025 Dec 15;14(12):1788. doi: 10.3390/biology14121788 (PMC12730348; doi:10.3390/biology14121788)
Supplement: Supplementary file 1 [file biology-14-01788-s001.zip › biology-3960981-supplementary.pdf]

The Mechanisms of Soil Conditioner and Switchgrass in Improving Saline–Alkali Soil: A Field Study in a Semi-Arid Area

Yixuan Li[1], Qing Liu[1], Longfei Kang[1], Kaiyu Zhang[1], Qiang Li *[1], Feng Ai[2]

Shaanxi Key Laboratory of Ecological Restoration in Northern Shaanxi Mining Area, Yulin University, Yulin, Shaanxi 719000, China

State Key Laboratory of Soil Erosion and Dryland Farming on the Loess Plateau, Institute of Soil and Water Conservation, Ministry of Water Resources and Chinese Academy of Sciences, Yangling, Shaanxi 712100, China

Yixuan Li, 15319957431@163.com

Qing Liu, 15529502972@163.com

Longfei Kang, kanglongfeishzu@163.com

Kaiyu Zhang, destiny789@yeah.net

Qiang Li, mr.li_qiang@163.com

Feng Ai,18717670799@163.com

*** Corresponding author**: Prof. Li Qiang*

Address: Shaanxi Key Laboratory of Ecological Restoration in Northern Shaanxi Mining Area, Yulin University, Yulin, Shaanxi 719000, China

Tel.: +86-181-6519-8826; E-mail:mr.li_qiang@163.com

Declaration: The authors declare no competing financial interest.

Note: Color should be used for all figures in print.

Table S1.Classification standards of saline–alkali soil

Grading Criteria	Non-salinized	Mild	Moderate	Severe	Salt soil
Total salt content (Total Dissolved Solids)	<0.1%	0.1-0.2%	0.2-0.4%	0.4-0.6%	>0.6%
	<1 g/kg	1-2 g/kg	2-4 g/kg	4-6g/kg	>6 g/kg

Table S2.Basic properties of soil conditioners

	Bulk density （g/cm²）	Large agglomerates（%）	Micro-agglomerates（%）	organic matter （g/kg）	pH	Alkaline hydrolyzable nitrogen （mg/kg）	Available phosphorus （mg/kg）	Fast-acting potassium （mg/kg）
Soil conditioner	0.65	67.8	32.2	230.17	7.24	138.32	46.92	2160

Table S3. Soil physicochemical indexes of different switchgrass varieties(The a b c d is difference was significant. *P*<0.05)

variety	Plan high/cm	leaf area/cm²	chlorophyllmg/g	Net photosynthetic rate/μmol m-²s-¹	CO_2 concentration/μmol mol-¹
YM-1	85.87±7.925 b	45.74±3.672	42.87±2.7570 a	5.83±0.0120 b	1.02±0.00033 b
YM-2	91.13±7.385 ab	51.25±11.969	38.87±1.9802 a	10.51±0.1155 a	1.03±0.00007 ab
YM-3	108.7±4.486 a	109.56±62.827	47.1±14.7815 a	5.75±0.0781 b	0.98±0.008847 d
YM-4	90.6±5.895 ab	42.09±9.210	40.27±5.3645 a	5.36±0.8521 b	1±0.0418 c
YM-5	107.47±5.060 ab	85.93±23.899	42.53±0.2603 a	11.37±0.7145 a	1.04±0.00351 a

Note: Values are expressed as mean ± standard error, and different letters in the same column indicate significant differences at the $p < 0.05$ level。
